# Effect of DrOnedarone on atrial fibrosis progression and atrial fibrillation recurrence postablation: Design of the EDORA randomized clinical trial

**DOI:** 10.1111/jce.15274

**Published:** 2021-11-02

**Authors:** Nassir F. Marrouche, Lilas Dagher, Oussama Wazni, Nazem Akoum, Moussa Mansour, Abdel Hadi El Hajjar, Arezu Bhatnagar, He Hua, the EDORA Investigators

**Affiliations:** ^1^ Department of Cardiology Tulane University School of Medicine New Orleans Los Angeles USA; ^2^ Department of Cardiac Electrophysiology Cleveland Clinic Cleveland Ohio USA; ^3^ Department of Cardiology University of Washington Medical Center Seattle Washington USA; ^4^ Department of Cardiology Massachusetts General Hospital Boston Massachusetts USA; ^5^ Department of Epidemiology Tulane University School of Public Health New Orleans Los Angeles USA

**Keywords:** ablation, atrial fibrillation, dronedarone, fibrosis

## Abstract

**Background:**

Atrial fibrillation (AF) recurrence after catheter ablation is associated with worse outcomes and quality of life. Left atrial (LA) structural remodeling provides the essential substrate for AF perpetuation. Baseline extent and the progression of LA fibrosis after ablation are strong predictors of postprocedural AF recurrence. Dronedarone is an antiarrhythmic drug proven to efficiently maintain sinus rhythm.

**Objective:**

We sought to investigate the effect of the antiarrhythmic drug Dronedarone in decreasing LA fibrosis progression and AF recurrence after ablation of AF patients.

**Methods:**

EDORA (NCT04704050) is a multicenter, prospective, randomized controlled clinical trial. Patients with persistent or paroxysmal AF undergoing AF ablation will be randomized into Dronedarone versus placebo/standard of care. The co‐primary outcomes are the recurrence of atrial arrhythmias (AA) within 13 months of follow‐up after ablation and the progression of left atrial fibrosis postablation. All patients will receive a late‐gadolinium enhancement magnetic resonance imaging at baseline, 3‐ and 12‐month follow‐up for the quantification of LA fibrosis and ablation‐related scarring. AA recurrence and burden will be assessed using a 30‐day ECG patch every 3 months with daily ECG recordings in between. Quality of life improvement is assessed using the AFEQT and AFSS questionnaires.

**Conclusion:**

EDORA will be the first trial to assess the progression of LA structural remodeling after ablation and its association with Dronedarone treatment and ablation success in a randomized controlled fashion. The trial will provide insight into the pathophysiology of AF recurrence after ablation and may provide potential therapeutic targets to optimize procedural outcomes.

## 1 CLINICAL BACKGROUND

The optimal treatment of atrial fibrillation (AF) continues to be a challenge and practices regarding its management are constantly evolving. While AF‐related symptoms and stroke risk can be managed using rate control strategies and anticoagulation, the recently published EAST‐AFNET trial^1^ emphasized the importance of early rhythm control for optimal outcomes such as decreased mortality, cardiovascular hospitalization, and stroke. Therefore, achieving sinus rhythm maintenance constitutes an important therapeutic target in AF patients. More recently, catheter‐based AF ablation procedures have been widely adopted as multiple clinical trials continue to highlight its efficacy in reducing AF recurrence and burden and in improving quality of life (QoL).^2^, ^3^, ^4^

Late Gadolinium Enhancement‐Magnetic Resonance Imaging (LGE‐MRI) represents an accurate and reproducible tool for assessing the extent of left atrial fibrotic remodeling.^5^, ^6^, ^7^, ^8^, ^9^, ^10^ The fibrotic tissue within LA myopathy generates a proarrhythmogenic substrate that can perpetuate re‐entrant circuits and automatic AF triggers.^11^ LA fibrosis has been shown to be a significant predictor of AF ablation success^5^ and major cardiovascular and cerebrovascular events in AF patients.^12^ The predictive value of LA fibrosis for postablation outcomes remains consistent through multiple observational and systematic review studies.^13^, ^14^, ^15^, ^16^, ^17^ While Akoum et al. reported that a higher fibrosis residual after ablation was significantly associated with AF recurrence, Kheirkhahan et al.^18^ showed that the extent of fibrosis progression after catheter ablation was also a strong predictor of ablation success rates. Therefore, reducing the progression of fibrosis after ablation constitutes a promising therapeutic target to optimize postablation outcomes.

Dronedarone, an antiarrhythmic drug with Vaughan‐Williams class I–IV properties, has been shown to be effective in reducing AF burden^19^, ^20^ and cardiovascular hospitalization rates^21^ in both placebo‐controlled trials and in real‐world studies^21^, ^22^, ^23^, ^24^ compared to other AADs. Additionally, Dronedarone exhibits anti‐remodeling properties^25^, ^26^ that could potentially help in the reduction of fibrosis progression after ablation, thus decreasing AF recurrence. Therefore, the EDORA trial (NCT04704050) aims to investigate the impact of Dronedarone on the progression of LA fibrosis and atrial arrhythmias (AA) recurrence after AF ablation therapy.

## 2 METHODS

### 2.1 Study design

**Figure 1:**
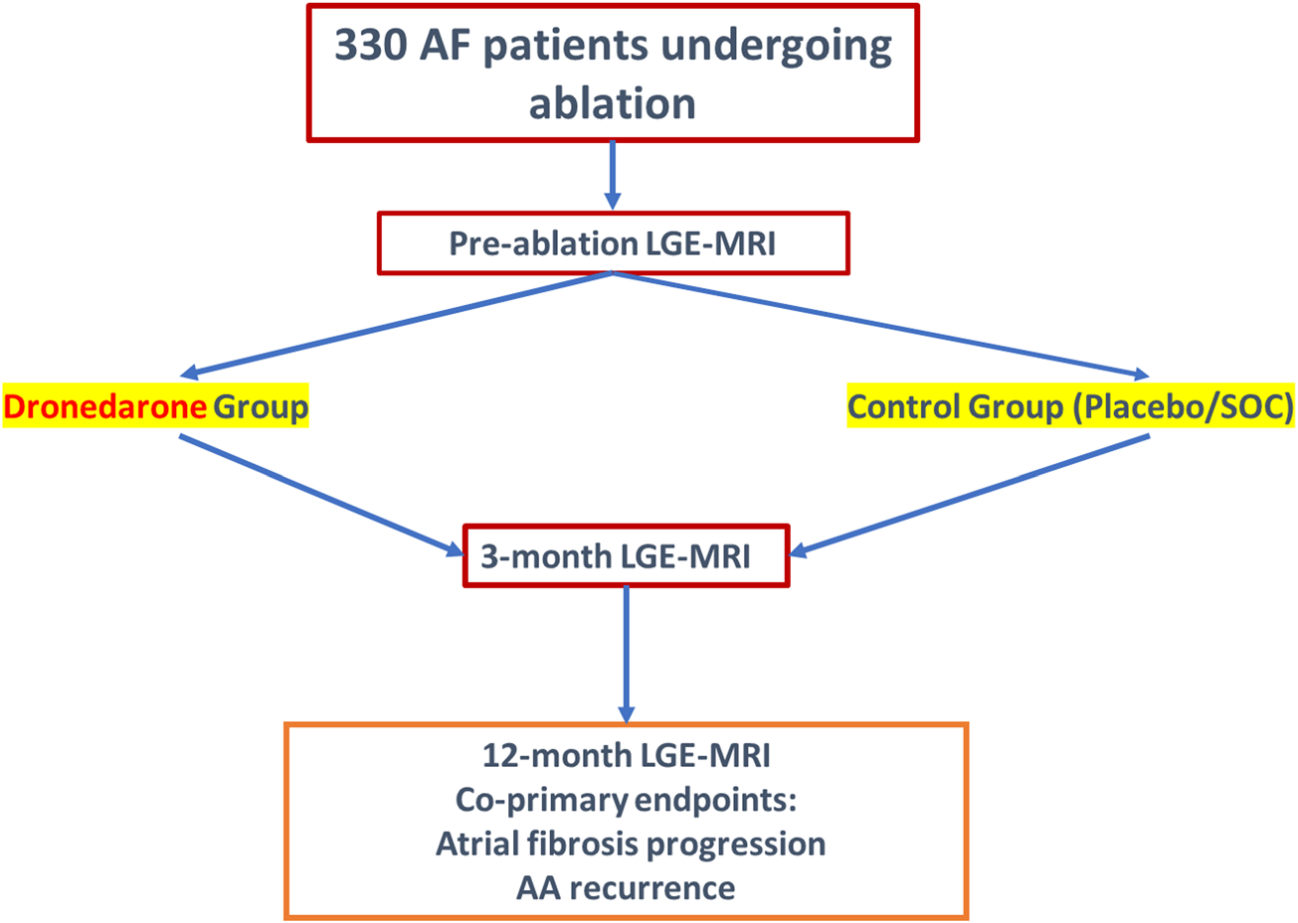
EDORA design flowchart. AA, atrial arrhythmia; AF, atrial fibrillation, LGE‐MRI, late gadolinium enhancement magnetic resonance imaging, SOC, standard of care

EDORA is a multicenter, prospective, phase IV, randomized controlled clinical trial. Patients with either paroxysmal or persistent AF undergoing first‐time ablation will be randomized in a 1:1 single‐blinded fashion into Dronedarone versus Control (placebo/standard of care) groups. In case of symptomatic arrhythmia recurrence in the control group, it will be left to the physician discretion to initiate rhythm control as part of standard of care. Patients will be followed for 13 months for the assessment of AA recurrence, as well as the progression of LA fibrosis after ablation. AA recurrence will be assessed using 30‐day ECG wearable patch every 3 months starting immediately after ablation, with daily ECG strip recordings performed by the patient using the patch in between continuous monitoring periods. The last 30‐day patch will be given to the patient at 12 months postablation, resulting in a total of 13 months of follow‐up. The endpoint of LA fibrosis progression will be evaluated using baseline, 3‐ and 12‐month LGE‐MRI. A flow chart of the study design is shown in Figure 1. The study will be performed across 15 centers with an estimated study population of 330 patients. Before trial initiation, sites must have a recruitment potential and the proper infrastructure for fibrosis imaging (ability to perform LGE‐MRIs). The study protocol will receive approval by the ethics review board at each site.

### 2.2 Patient population

Inclusion criteria in our trial are the following: (1) Male or female patients aged ≥18 years; (2) Patients with paroxysmal or persistent AF who are undergoing their first ablation of AF, regardless of whether they were receiving an AAD before enrollment or not. Key exclusion criteria include contraindications to Dronedarone; contraindications to gadolinium or MRI; liver or lung toxicity; pregnancy, or severe renal disease (with eGFR <30 ml/min). A full list of eligibility criteria are shown in Table 1. Informed consent will be obtained from all patients before enrollment.

**Table 1:** Eligibility criteria for the EDORA trial

**Inclusion criteria**
Patients with paroxysmal or persistent AF
Undergoing first AF ablation, regardless of whether they were receiving an AAD before enrollment or not.
Age ≥18 years
**Exclusion criteria**
Any health‐related gadolinium/MRI contraindications (e.g., allergy to gadolinium, pacemakers, Implantable Cardioverter Defibrillators [ICD's], other devices/implants contraindicated for use of MRI, etc.).
Patients weighing >300 lbs. (MRI quality decreases as BMI increases).
Patients with contraindications to Dronedarone: Patients with decompensated heart failure or class NYHA IV, second or third‐degree atrioventricular (AV) block or sick‐sinus syndrome (except when used in conjunction with a functioning pacemaker), concomitant use of strong CYP‐3A inhibitors or other Class I or III AADs, Drugs or herbal products that prolongs the QT interval and may induce Torsade de Pointes Liver or lung toxicity related to the previous use of amiodarone, Severe hepatic impairment including any stage of cirrhosis and acute liver failure Bradycardia <50 bpm, QTc Bazett interval ≥500 ms or PR interval >280 ms Hypersensitivity to the active substance or to any of its excipients
Acute or chronic severe renal disease with a low glomerular filtration rate (GFR), < 30 ml per minute per 1.73 m^2^
Patients with a history of prior left atrial ablation or valvular cardiac surgery (myocardial scarring/fibrosis from prior surgeries may confound data) premenopausal (last menstruation ≤1 year before screening) who: a. Are pregnant or breast‐feeding or plan to become pregnant during the study period or, b. Are not surgically sterile or, c. Are of childbearing potential and not practicing two acceptable methods of birth control or, d. Do not plan to continue practicing two acceptable methods of birth control throughout the trial (highly effective methods of birth control are defined as those, used alone or in combination, that result in a low failure rate, that is, less than 1% per year when used consistently and correctly).
Patients who do not have access to the Internet/e‐mail.
Patients with cognitive impairments who are unable to give informed consent.

### 2.3 Outcomes

The study has two co‐primary endpoints. The first co‐primary endpoint will be the time to first recurrence of AA. AA (AF, atrial flutter, or atrial tachycardia) recurrence is defined by the first episode lasting >30 s, or by a new AAD initiation after randomization for AA recurrence after AF ablation, including early recurrence during the initial 90 days postprocedural “blanking period.” The inclusion of the “blanking period” in the assessment of the primary endpoint of AA recurrence is based on multiple reasons. First, there is a lack of consensus and definitive data confirming the clinically relevant duration of the blanking period.^27^ Additionally, early AA recurrences can still cause higher hospitalization rates, lower QoL, and an increased risk of later AA recurrence.^28^, ^29^

The second co‐primary endpoint will be the progression of LA fibrosis after ablation. LA fibrosis progression is determined by the difference in percentage of new fibrosis seen on the 12‐month LGE‐MRI, compared to the baseline and 3‐month LGE‐MRI. A more detailed protocol is outlined below.

Secondary outcomes of the study include AA burden, AAD initiation change or adjustment, incidence of symptomatic AA episodes, repeat ablation, cardioversion, QoL assessment, and LA and LV function assessment. Exploratory outcomes include cardiovascular hospitalization, cardiovascular mortality, and stroke/TIA. Secondary endpoints will be analyzed at 0–3, 3–13, and full 13‐month (0–13) postablation and compared between the two trial arms.

Safety outcomes will also be assessed throughout the follow‐up period and include any adverse event related to AADs. Patients will be evaluated at their 3‐ and 12‐month visits, as well as during their 6 and 9‐month phone calls for safety outcomes using unsolicited general health questions. A complete liver panel drawn at baseline and 3‐month follow‐up will assess for liver toxicity. A list of all assessed safety outcomes is shown in Table 2.

**Table 2:** List of safety outcomes to be monitored

Adverse events related to AAD treatment
Increase in renal creatinine
Pulmonary toxicity
Thyroid toxicity
Hepatic toxicity
Bradycardia <50 bpm
QT prolongation
Gastrointestinal side effects (dyspepsia, diarrhea, nausea, vomiting)
Heart failure or decompensations in HF patients

### 2.4 Randomization and blinding

Patients who meet the eligibility criteria will be randomly assigned in a 1:1 ratio into Dronedarone versus control group after undergoing their AF ablation procedure using randomization software. Randomization will be stratified by age (< or ≥65 years old) and type of AF (persistent vs. paroxysmal). This will be a single‐blinded study: only patients will be blinded to the treatment administered to prevent bias in assessing QoL and symptoms. Participating physicians will not be blinded to group assignment to prevent unnecessary discontinuations of treatment that can impact the co‐primary endpoint of fibrosis progression. Additionally, all analysts reviewing LGE‐MRI images and ECG strips will be blinded to patient's arm assignment.

### 2.5 Intervention

The treatment group will receive Dronedarone 400 mg to be taken orally twice daily throughout the 13‐month follow‐up period. Patients randomized to the Dronedarone group will receive an initial dose after cardiac ablation. If AAD change or discontinuation occurs, the patient will continue to be monitored for fibrosis progression and AF recurrence and burden postablation throughout the duration of the trial.

The control group will receive a matched dose of placebo, starting with the initial dose after ablation. In the case of AF recurrence, initiation of AADs will be left at the discretion of the treating physician, with recommendations to limit the use of AADs to necessary cases only and to avoid prescribing amiodarone or Dronedarone. In the case of a new AAD initiation in the control arm, placebo treatment will be discontinued. The patient will continue to be monitored for fibrosis progression after ablation using LGE‐MRI scans, and AF burden.

### 2.6 AF ablation procedure

All AF ablation procedures will be performed by experienced cardiac electrophysiologist in accordance with the latest guidelines.^30^, ^31^ In summary, pulmonary veins will be electrically isolated by creating lesions around the pulmonary veins (PV) antra. Entrance block in all pulmonary veins will be confirmed using standard techniques.^30^ If normal sinus rhythm could not be restored at the end of the pulmonary vein isolation, and despite cardioversion, performing additional lesion sets will be left at the discretion of the operator to eliminate the arrhythmia. The technique to be used for the AF ablation procedure (radiofrequency or cryoballoon) will also be left at the discretion of the operator. Information about ablation techniques and performed lesions sets will be collected, and will be accounted for in further analyses of the trial's results.

### 2.7 Imaging protocol

#### 2.7.1 Baseline LGE‐MRI to quantify baseline atrial fibrosis

Quantification of LA fibrosis will be obtained using methods previously described.^5^ The LA wall will be segmented manually and regions of fibrosis in LGE‐MRI images will be defined by an intensity threshold determined by expert inspection. Fibrotic tissue is detected when its enhancement is one‐to‐five standard deviations above the mean of normal tissue intensity. A 3D LA fibrosis map will be created using Corview Volume Rendering Software (Marrek Inc.). Interobserver and intraobserver reproducibility for these techniques have been previously reported.^6^, ^7^ Fibrosis will be represented as the volumetric percentage of left atrial wall enhancement, as well as in total volume (cm^3^).

#### 2.7.2 Three‐month LGE‐MRI to visualize ablation‐induced scars

Quantification of ablation‐induced scarring in the LA with LGE‐MRI has been previously described.^6^, ^32^ Three months after the ablation, endocardial and epicardial borders of the LA wall are contoured manually and blood pooling or other artifacts are omitted in scanned images. Pixel intensities are distributed as bimodal to distinguish normal from injured tissue. Pixels with lower intensities are chosen as normal tissue. Ablation‐induced lesions are defined at >3 SD above blood pool mean, as done in other studies^6^, ^33^, ^34^ and in accordance with histologically validated data.^35^ Regions marked as the ablation lesion will be independently evaluated by two blinded experts to ensure correctness.

#### 2.7.3 Twelve‐month LGE‐MRI to assess for new fibrosis formation

After segmentation and measurement of LA fibrosis and scarring on preablation scans and on 3‐month postablation scans, respectively, we will use the latter as baseline for our trial to see if any new enhancement has formed or regressed. Transient postablation lesions will be defined as enhancements detected on the first postablation scan but not in the second postablation scan. New fibrosis will be defined as enhancement detected on the second postablation scan and absent on the first postablation scan. Both of these parameters will be reported as the percentage of total LA wall volume.

### 2.8 Follow‐up

Subjects will receive an FDA‐approved 30‐day ECG wearable patch (BodyGuardian® MINI PLus, Preventice). Participants will be instructed to wear it once every 3 months, including month 12 to obtain a 30‐day continuous ECG strip, starting with immediate use one day after ablation. The patch will also allow patients to record daily ECG strips in between the 30‐day continuous monitoring periods, and at any AF‐related symptom occurrence throughout the trial. This technology provides the advantage of being a reliable tool to continuously monitor heart rhythm without being invasive. The ECG Core lab at the University of Washington, WA will be responsible for reviewing and analyzing all ECG patch recordings. All analysts will be blinded to patient's assigned group. Additionally, QoL will be assessed at baseline, 3 and 12 months follow up using the AFEQT and AFSS questionnaire. Follow‐up phone calls will be scheduled around the 6‐ and 9‐month mark of the trial by a study coordinator. Compliance to treatment, as well as the maintenance of the wearable patch will be assessed at that time.

**Figure 2:**
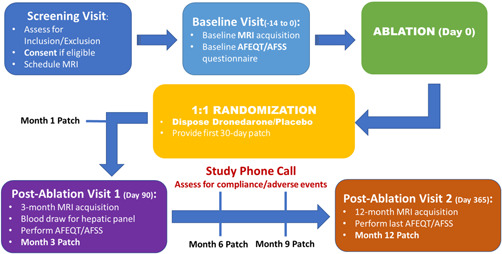
Follow‐up design for the EDORA trial. AFEQT, atrial fibrillation effect on QualiTy‐of‐life, AFSS, Atrial Fibrillation Severity Scale; MRI, magnetic resonance imaging

The follow‐up schedule is summarized in Figure 2.

### 2.9 Sample size estimation

For the two co‐primary endpoints:

#### 2.9.1 Atrial fibrosis progression

Based on the assumption that the mean progression of fibrosis in the control group is 10%, with a standard deviation of 1.59%. Predicting a 20% dropout rate, 86 randomized patients will provide >99% power with a two‐sided *α* = .05 to detect a reduction of 20% in fibrosis progression between dronedarone and the control arm. Given that an estimated 330 patients will be recruited, the study will be adequately powered to detect any group difference in atrial fibrosis progression.

#### 2.9.2 AF recurrence

Based on the following assumptions:

- 50% of patients in the control group will have AA recurrence in 1‐year, postablation (including during the blanking period) and,
- the relative reduction of AA recurrence will be by 33% in the Dronedarone group compared to the control group and,
- 20% of subjects could be lost during the follow‐up period and,
- two‐sided *α* = .05.

the EDORA trial will require a total of 330 patients participating, thus, 165 in each trial arm. Therefore, a calculated total of 138 AF recurrences will have 86% power to detect any group differences.

### 2.10 Statistical analysis

Descriptive statistics such as mean (SD) for continuous variables and frequency (%) for categorical variables will be provided for baseline information. The Student *t* test/Wilcoxon rank‐sum test or chi‐square tests will be applied to compare the demographic information and the baseline clinical measurements between the intervention and control groups to assess if randomization is successful at each site. Any imbalanced information will be included in the regression models to account for potential confounding variables in assessing treatment effects. All primary efficacy analysis will be performed in an intent‐to‐treat (ITT) manner. All statistical tests will be two‐sided with a *p* ≤ .05. Time‐to‐event‐analysis will be conducted for AA recurrence. Cumulative AA rates will be calculated by intervention conditions using the Kaplan–Meier (KM) method and compared using the log‐rank test. Therefore, we will first investigate the distribution of the outcome, and if the distribution assumption is reasonable, we will use linear mixed model, otherwise, we will use generalized estimating equation approach. The study is defined as positive if the analyses yield the coefficients of the intervention for both co‐primary endpoints with a right direction and a *p* < .05.

## 3 DISCUSSION

Investigations regarding the pathophysiology of AF have paved the way for novel therapeutic targets. Indeed, a larger amount of evidence shows that AF does not originate solely from the sleeves of the PVs, but also from an arrhythmogenic substrate generated by the deposition of fibrotic tissue in the interstitial space of the LA myocardium.^11^, ^36^ LA fibrosis, a hallmark of LA myopathy, has been shown to sustain abnormal electrical activity and the formation of re‐entrant circuits through multiple mechanisms, including Ca signaling dysregulation and abnormal cardiomyocyte–fibroblast coupling, leading to AF generation and maintenance even after a durable PVI ablation.^11^, ^36^ In multicenter observational studies, LA fibrosis at baseline proved to be a strong independent predictor of AF recurrence after ablation. Every 1% increase in baseline atrial fibrosis was shown to be associated with 6% increased risk of AF recurrence after ablation.^5^ Akoum et al.^13^ found that only the residual fibrosis (not targeted by ablation during index procedure) after ablation correlated independently with AF recurrence, regardless of the number of PV encircled. Furthermore, LA structural remodeling remains a dynamic process, as data demonstrates the progression or formation of new fibrosis after ablation,^18^ possibly induced by ongoing inflammatory processes. Each 1% increase in fibrosis levels after ablation was associated with a 3% risk of AF recurrence.^18^ In addition to its correlation with ablation success rates, LA fibrosis has also been shown to predict major cardiovascular and cerebrovascular events.^12^ Therefore, targeting atrial fibrosis progression after ablation may constitute a novel therapeutic strategy to optimize ablation success rates, and possibly improve long‐term hard outcomes.

During episodes of AF, factors known to induce atrial fibrosis growth such as, collagen‐1 and fibronectin‐1, are released into atrial tissues.^37^ For this reason, rhythm control by means of an efficient AAD may serve as a way to limit the amount of time available for these biomarkers to disperse into atrial tissues as well as, proactively managing fibrosis progression. The antiarrhythmic drug Dronedarone showed a clinical benefit in decreasing mortality and cardiovascular hospitalizations in the ATHENA trial.^21^ Dronedarone has also proven to be efficient in maintaining sinus rhythm in AF patients in EURIDIS/ADONIS,^19^ HESTIA,^20^ and DAFNE trials,^22^ but no trial investigated its efficacy in a postablation population. Additionally, regardless of sinus rhythm maintenance, dronedarone exert vasodilatory properties, with effects on coronary and cerebral blood flow in animal models, and possible anticoagulation/antiplatelet effects.^38^, ^39^, ^40^ In a subanalysis of the ATHENA trial,^21^, ^41^ Dronedarone decreased the risk of stroke by 34% compared to placebo, and this effect remained consistent regardless of sinus rhythm maintenance. These observations could suggest that Dronedarone has the potential to impact LA structural remodeling, with a potentially lower risk of thromboembolism, independent of its antiarrhythmic properties. In fact, in addition to its anti‐adrenergic (class II) and vasodilatory (class IV) effects, Dronedarone may exhibits anti‐inflammatory and anti‐fibrotic properties that could modify the arrhythmogenic substrate of the LA. One of the main speculated mechanisms of action has been derived from preclinical publications and is based on Dronedarone preventing vascular alterations that participate in structural remodeling and atrial maintenance substrate. Reactive oxygen species (ROS) generated in the myocardium promotes endothelial to mesenchymal transition (EndMT) by reducing endothelial nitric oxide (NO) production.^42^ Numerous studies have shown that ROS and EndMT are major contributors towards cardiac fibrosis.^43^ Circulating symmetric dimethylarginine (SDMA), a biomarker that reduces the synthesis of NO,^44^ is also associated with increase in atrial wall thickness in AF. Dronedarone stimulates nitric oxide synthase (NOS) which increases the bioavailability of NO,^45^ and reduces SDMA,^46^ playing a critical role in preserving the epithelial phenotype. In fact, there is an improvement in global antioxidant status after treatment with Dronedarone.^46^ Furthermore, a decrease in collagen deposition in the atria has been observed after treatment with Dronedarone,^26^ thereby improving atrial structure and decreasing wall thickness.

As the role of fibrosis in maintaining AF becomes pivotal, the EDORA trial will be the first randomized controlled trial to investigate LA fibrosis progression as a main therapeutic target after ablation using an AAD. EDORA will also be the first trial to assess the efficacy of Dronedarone in a postablation population in decreasing AF recurrence. The impact of ablation on LA remodeling parameters have been reported previously in acute and sub‐acute settings,^47^ but data on its long‐term impact on structural and functional remodeling of the LA remain scarce. Using advanced cardiac images at baseline and follow‐up, this trial will provide new insights regarding changes in the LA induced by the ablation up to 12 months after the procedure, as well as strengthen our understanding on chronic lesion formation and factors that may influence the durability of ablation lesions and PVI. Finally, the EDORA trial makes use of a wearable ECG monitoring device to provide valuable information on AF burden and possibly refine the definition of ablation success, as the 30 s threshold for an AF episode indicating ablation failure remains controversial.^48^ Exploring the association between AF burden and LA structural remodeling will also allow to better understand the mechanistic process linking both entities.

## 4 CONCLUSION

EDORA is the first prospective, multicenter, randomized clinical trial investigating Dronedarone in AF patients after catheter ablation and its impact on LA fibrosis progression and AF recurrence. The trial will provide insight into the pathophysiology of AF recurrence after ablation and may provide potential therapeutic targets to optimize procedural outcomes.

## CONFLICT OF INTERESTS

Dr. Marrouche reports having received consulting fees from Biosense Webster, as well as research funding from Biosense Webster, Abbot, Medtronic, and Boston Scientific. Dr. Wazni reports receiving consultant fees from Boston Scientific, Medtronic, Biosense Webster. All other authors report no conflict of interest.

## Data Availability

Data sharing is not applicable to this article as no new data were created or analyzed in this study.
